# Validity and Reliability of the Korean Version of the Pregnancy Physical Activity Questionnaire

**DOI:** 10.3390/ijerph17165873

**Published:** 2020-08-13

**Authors:** Jeong-Won Han, Ji-Soon Kang, Hanna Lee

**Affiliations:** 1 College of Nursing Science, Kyung Hee University, Seoul 02447, Korea; hjw0721@khu.ac.kr; 2 Department of Nursing, Hansei University, Gunpo-si 43742, Korea; lemueljs@hansei.ac.kr; 3 Department of Nursing, Gangneung-Wonju National University, Wonju-si 26403, Korea

**Keywords:** exercise, pregnancy, reproducibility of results, women

## Abstract

The purpose of this study was to translate the Pregnancy Physical Activity Questionnaire, a semi-quantitative tool that asks participants about time spent on 32 activities, into Korean and verify its validity and reliability. In total, 363 pregnant women under prenatal care at an obstetrics and gynecology hospital and a postpartum care facility in Gyeonggi-do completed the Korean version of the Pregnancy Physical Activity Questionnaire. The questionnaire’s content validity, construct validity, concurrent validity, and reliability were verified. After verifying the validity of the contents of the Pregnancy Physical Activity Questionnaire, all the questions were included in the Korean version. For construct validity, we divided the participants into primipara and multipara groups based on their delivery history. On comparison of the two groups’ physical activity based on the responses to the Pregnancy Physical Activity Questionnaire, there was a statistically significant difference in the total activity (t = −4.56, *p* < 0.001) and the total activity (light activity or more) (t = −5.80, *p* < 0.001). The correlation between the Pregnancy Physical Activity Questionnaire and the Global Physical Activity Questionnaire was tested to establish concurrent validity, and a significant correlation was found between all items except for vigorous physical activity. The Guttmann reliability coefficient by the odd-even method was 84. The Korean version of the Pregnancy Physical Activity Questionnaire is a suitable tool to measure the physical activity of pregnant women and can be used in clinical practice.

## 1. Introduction

The benefits of physical activity during pregnancy include improved physical fitness and reduced risks of excessive weight gain, pre-eclampsia, and pre-term birth. During pregnancy, women experience involuntary physical and mental changes, and the maternal physiological system is transformed to embrace fetal growth and development [1,2]. According to a recent statistical report, the neonatal mortality rate in Korea is below the Organization for Economic Cooperation and Development (OECD) average at 2.8 per 100 births; the stillbirth rate, however, is higher than the OECD average, at 7.8 per 100 births [3]. Further, the rapid decline in birth rate, increased average age of the primipara, and the increased incidence of multiple births and preterm births threaten maternal health [4].

Although it may vary by national beliefs and characteristics, traditionally, adequate nutritional intake and rest have been prioritized for pregnant women [5]. Recently, however, moderate exercise has been suggested to help pregnant women adjust to the various physical, psychological, and social changes that occur from conception to delivery [6]. Exercising during pregnancy helps maintain a stable psychological state, and not only alleviates the stress, anxiety, and depression symptoms that occur during pregnancy [2], but also reduces the duration and pain of labor and facilitates postpartum recovery [7].

Despite such benefits, many social and cultural beliefs surrounding pregnancy complicate a woman’s choice to exercise [6]. Thus, most women choose to discontinue exercising once they discover that they are pregnant [8]. In Korea, many pregnant women avoid physical activities due to traditional ideas in Korean culture; others reduce activity as much as possible and seek stability and rest, which has led to significantly reduced physical fitness among women after pregnancy and childbirth [9]. In recent years, an appropriate level of physical activity and weight management have been strongly encouraged for pregnant women; this ensures the physical health of the mother, as well as that of the unborn child, and studies have even confirmed that they offer a source of pleasure during pregnancy [10,11,12]. A meta-analysis of the impact of physical activity in pregnant women reported that moderate-intensity activities lower excessive pregnancy weight gain, pregnancy diabetes, and postpartum depression [11]. Furthermore, a Canadian cohort study that examined the association between preconception body mass index (BMI) and pregnancy complications from 2004 to 2012 observed that differences of 20–30% from the preconception BMI increase the risk for caesarean section, shoulder dystocia, and neonatal intensive care unit (NICU) admission [10]; this highlights the need for efforts to boost physical activity among pregnant women.

Currently, diverse studies pertaining to physical activities for pregnant women are ongoing in a number of countries. In the United States, one study implemented and assessed the effects of a walking intervention for pregnant women using an activity monitor [12]. In Spain, a study investigated the effects of a short-term exercise program for pregnant women on weight gain during pregnancy [13]. In such cases, a device attached to the body measured the outcome variable for physical activity in pregnant women [12]; however, this device had shortcomings, such as the need for continuous attachment even during physical activity and device errors that hindered accurate measurements. Moreover, most physical activity questionnaires, such as the International Physical Activity Questionnaires (IPAQ) [14], do not include activities at home or those related to caregiving, which account for the majority of activities during pregnancy [15]. Questionnaires that assess physical activity during pregnancy must be capable of measuring a smaller range of physical activities than those targeting the general population. Accurately measuring physical activity in pregnant women is essential to understanding and providing recommendations for physical activities in pregnant women, addressing aspects such as drastic weight change during pregnancy and the consequent impact on health, patterns of weight management, and women’s lifestyles.

The Pregnancy Physical Activity Questionnaire (PPAQ) is an instrument recently developed by Chasan-Taber, Schmidt, Roberts, Hosmer, Markenson, and Freedson [16] that measures physical activity in pregnant women. The PPAQ is a semi-quantitative questionnaire that inquires about the duration of participation in various physical activities, including household tasks/caregiving (13 items), work (5 items), sports/exercise (9 items, including 2 open-ended questions), transportation (3 items), and rest (3 items). The questionnaire has addressed the limitations of existing instruments that were used to measure physical activity in pregnant women. In particular, in addition to including home or childcare-related activities, which account for a substantial portion of physical activities in pregnant women, the PPAQ has been confirmed to have a high validity and reliability for measuring the overall physical activity, spanning work, leisure, and hobbies. To effectively and continuously promote physical activities in pregnant women in Korea, accurately understanding physical activity and exploring tailored exercise interventions are crucial. However, there is virtually no Korean instrument available to accurately measure physical activity in pregnant women. Hence, this study aims to adapt the PPAQ into Korean and test its validity and reliability so as to present foundational data to explore physical activity interventions for pregnant women.

## 2. Materials and Methods

### 2.1. Research Design

This methodological study was conducted in order to validate the Korean version of the PPAQ, originally developed by Chasan-Taber et al. [16].

### 2.2. Participants

The participants were convenience sampled from pregnant women who were receiving prenatal care at an obstetrics/gynecology hospital in Gyeonggi Province. The inclusion criteria were women between the ages of 20 and 48, those who provided informed consent to participate, and those who were able to read and understand Korean without difficulty. The exclusion criteria based on exercise guidelines for pregnant women [9] included those with diabetes who required insulin injections, those with hypertension or heart disease who were taking medications, those with chronic kidney disease, those who had conceived twins, and those who had participated in a similar study in the past. The sample size required to test the validity and reliability of the instrument was calculated as follows. The minimum sample size required for validity testing was at least five times greater than the minimum number of items, and to ensure stable testing, at least 10 times greater than the number of items. Hence, for the 36 items on the PPAQ, at least 180—or 360 for more stable testing—participants are needed [17].

Further, we used the known-groups technique for construct validity testing, which involves administering the technique groups anticipated to differ in the construct of the study due to known characteristics. A previous study on pregnant women [18] confirmed that the amount of physical activity varies according to the presence of another child; based on this result, we classified the participants into groups according to the presence of other children. For the comparison of means between the two groups using independent t-tests with an alpha of 0.05, an effect size of 0.50, and a power of 0.90, as suggested by Cohen, the required minimum sample size was 84 for each group for a total sample size of 164 or greater. Based on this calculation, the final sample size of this study (n = 363) was deemed appropriate.

### 2.3. Measurement

#### 2.3.1. Pregnancy Physical Activity Questionnaire (PPAQ)

The PPAQ is a semi-quantitative questionnaire containing 36 items inquiring about the date of the questionnaire, the first day of the last period, the estimated due date, household tasks/caregiving (13 items), work (5 items), sports/exercise (9 items, including 2 open-ended questions), transportation (3 items), and rest (3 items). The participants were instructed to choose the category of duration that best represents the time they spent on the corresponding activities per day or week. Thirty-three items related to physical activity consisted of six categories with varying sub-categories depending on the item, and the participants chose an amount from 0 h per day or week to 6 or more hours per day or week. The reliability was assessed using intraclass correlation coefficients (ICC) from the study by Chasan-Taber et al. [16], with values of 0.82 for moderate-intensity activity, 0.81 for vigorous-intensity activity, 0.83 for sports/exercise, and 0.93 for work.

The PPAQ uses the physical activity equation suggested by Chasan-Taber et al. [16], where physical activity is calculated by multiplying the duration of each of the 33 physical activities by the average weekly energy expenditure (MET-h, week-1). The metabolic equivalent of task (MET) is the objective measure of the ratio of the rate at which a person expends energy, relative to the mass of that person, while performing some specific physical activity compared to a reference, set by convention at 3.5 mL of oxygen per kilogram per minute [19]. To calculate this, the duration of physical activity is first computed. The scores for the duration of activities in items 4–11, 14–16, and 20–22 are 0, 0.12, 0.50, 1.0, 2.0, and 3.0, which are multiplied by 7 to convert into weekly data. The scores for the duration of activities in items 12, 13, 32, 33, 34, 35, and 36 are 0, 0.12, 0.50, 2.0, 4.0, and 6.0, which are multiplied by 7 to convert them into weekly data. The scores for the duration of activities in items 17, 18, 19, 23, 24, 25, 26, 27, 28, 29, 30, and 31 are 0, 0.12, 0.50, 1.0, 2.0, and 3.0, and the scores are already in per-week units.

Second, the intensity of the average weekly energy expenditure is calculated by multiplying the MET value corresponding to each physical activity item: #4 = 2.5, #5 = 2.0, #6 = 3.0, #7 = 2.7, #8 = 4.0, #9 = 3.0, #10 = 4.0, #11 = 1.8, #12 = 1.0, #13 = 1.1, #14 = 3.2, #15 = 2.3, #16 = 2.3, #17 = 2.8, #18 = 2.8, #19 = 4.4, #20 = 2.5, #21 = 4.0, #22 = 1.5, #23 = 3.2, #24 = 4.6, #25 = 6.5, #26 = 7.0, #27 = 3.5, #28 = 6.0, #29 = 4.5, #32 = 1.6, #33 = 3.0, #34 = 2.2, #35 = 4.0, #36 = 3.3. For open-ended items 30 and 31, the participants’ responses are classified into sedentary activity (<1.5 METS), light-intensity activity (1.5–<3.0 METS), moderate-intensity activity (>3.0–<6.0 METS), and vigorous-intensity activity (>6.0 METS), and are weighted accordingly.

Third, total activity is the sum of the duration and intensity of all activities and is calculated using the following equation: total activity = sum (duration × intensity) (items #4–36). Total activity (≥light-intensity activity) = sum (duration × intensity) (items #4, 5, 6, 7, 8, 9, 10, 11, 14, 15, 16, 17, 18, 19, 20, 21, 22, 23, 24, 25, 26, 27, 28, 29, 32, 33, 34, 35, 36, and #30 or 31 with >1.5 METS).

Fourth, the PPAQ classifies physical activity into four types of intensity (sedentary, light, moderate, and vigorous), and the equations used for each type are as follows. Sedentary activity (number of items = 2 + 2 open-ended item) = sum (duration × intensity) (items #12 and 13, and #30 or 31 with <1.5 METs). Light-intensity activity (number of items = 12 + 2 open-ended item) = sum (duration × intensity) (items #4, 5, 7, 11, 15, 16, 17, 18, 20, 22, 32, 34, and #30 or 31 with 1.5–<3.0 METs). Moderate-intensity activity (number of items = 15 + 2 open-ended item) = sum (duration × intensity) (items #6, 8, 9, 10, 14, 19, 21, 23, 24, 27, 28, 29, 33, 35, 36, and #30 or 31 with >3.0–<6.0 METs). Vigorous-intensity activity (number of items = 2 + 2 open-ended items) = sum (duration × intensity) (items #25, 26, and #30 or 31 with >6.0 METs).

Fifth, the PPAQ classified physical activity according to five types of activities (household tasks/caregiving, occupational sports/exercise, transport, inactivity), and the following equations are used. Household tasks (number of items = 13) = sum (duration × intensity) (items #4, 5, 6, 7, 8, 9, 10, 14, 15, 16, 17, 18, 19. Work (number of items = 5) = sum (duration × intensity) (items #32, 33, 34, 35, 36. Sports/exercise (number of items = 7 + 2 open-ended) = sum (duration × intensity) (items #23, 24, 25, 26, 27, 28, 29, 30, 31. Transport (number of items = 3) = sum (duration × intensity) (items #20, 21, 22). Inactivity (number of items = 3) = sum (duration × intensity) (items #11, 12, 13).

#### 2.3.2. Global Physical Activity Questionnaire (GPAQ)

GPAQ was utilized to test concurrent validity in this study. The GPAQ is a standardized questionnaire developed by the World Health Organization (WHO) in 2002 to compare the level of physical activities across countries; it is currently utilized in 50 countries [20]. The GPAQ consists of 16 types of activities in three domains (work, transport, leisure) to measure the activity intensity, duration, and frequency, and the MET energy consumption (MET∙min∙wk) is calculated by weighting the product of the duration of activity per week (min) and the frequency (number of activities) with the intensity of activity (vigorous, moderate). In the present study, the Korean version—Global Physical Activity Questionnaire (K-GPAQ) was used, and the reliability of the instrument, as measured with ICC, ranged from 0.62 to 0.70.

The GPAQ broadly consists of three domains (work, transport, and leisure), and physical activity is calculated by multiplying the number of days of physical activity by the duration of the physical activity in minutes using the following equation: vigorous-intensity activity = (number of days of vigorous work-related activity × time) + (number of days of vigorous leisure activity × time); moderate-intensity activity = (number of days of moderate work-related activity × time) + (number of days of transport × time) + (number of days of moderate leisure activity × time).

### 2.4. Data Collection

The questionnaires were distributed to pregnant women who were convenience sampled from those who visited the outpatient clinic of S Women’s Hospital (250 deliveries per month) or A Women’s Hospital (300 deliveries per month) in K for prenatal care, and those who are undergoing prenatal care (prenatal education) at A postpartum care facility. The data were collected from 1 July to 26 September, 2019. We met with the directors of the hospitals and the nursing department to explain the purpose and aim of the study. Permission was obtained prior to data collection. Before administering the questionnaire, we adequately informed the prospective participants about the purpose and method of the study and obtained written consent from those who were willing to participate. They were informed using easy language about the purpose of the study and that the collected data would only be used for research purposes, all personal information would only be used in identifying the data, and the participants’ names would remain anonymous in the actual analysis for statistical processing.

These participants were given a structured self-reported questionnaire with a small gift and instructed to complete the questionnaire themselves. The data were collected outside the hours of their prenatal care so as not to interrupt their care, and the participants were also informed that they could withdraw from the study at any time and request the deletion of some or all of the contents as they wished. Furthermore, as this study was conducted on pregnant women, who are a vulnerable group of individuals, we appointed an expert on rights and welfare for consultation about research ethics. In addition, we notified the participants of matters stipulated by the Bioethics and Safety Act.

### 2.5. Ethical Considerations

This study was approved by the institutional review board at an institution in C city (1,040,271-201,905-**-039). Although this study was conducted on pregnant women, the study was judged to have minimal risk from IRB because it is a non-interventional study and survey.

### 2.6. Data Analysis

In this study, we finalized the Korean version of the instrument through a process of forward translation, a review by an expert panel, back translation, and cognitive evaluation based on the WHO guidelines for the translation and cross-cultural adaptation of instruments [21]. Prior to the first round of translation, we obtained permission to translate and use the instrument in Korean from the original developers. The first round of translation was independently performed by two nursing PhDs who are fluent in English and are adequately experienced with professional nursing terminology. The two translators agreed on the final translations, such that literal translations are avoided and the meanings of the sentences are well conveyed, as opposed to translating each word. Then, a panel of experts, consisting of a professional bilingual translator, two nursing professors proficient in both languages, and the two nursing PhDs who performed the first round of translation, reviewed the translation against the English source. The agreement and precision of the sentences were reviewed, and some were modified and adapted to the Korean culture in consideration of readability and cultural differences.

After this process, an American native English speaker who had no prior experience with the tool and is proficient in Korean performed a back-translation. After confirming that the back-translated sentences were not the same as the source but did not have any meaning changes, we finalized the first draft of the translated instrument. The content validity of the instrument was reviewed by one Ob/Gyn specialist, two nursing PhDs, two nursing professors, and one nursing PhD student with more than five years of clinical experience. The validity of each item was tested using the content validity ratio (CVR), the degree of convergence, the degree of consensus, and the coefficient of variation. After establishing content validity, a pilot test was conducted on three pregnant women to examine the readability and comprehensibility of the instrument and make necessary revisions. The collected data were analyzed using IBM Statistics AMOS 22.0 and the SPSS Statistics 20 software. The participants’ demographics were analyzed using frequency, percentage, mean, and standard deviation.

To test the construct validity of the Korean version- Pregnancy Physical Activity Questionnaire (K-PPAQ), the participants were divided into two groups depending on the presence of other children, and the differences in physical activities between the groups were analyzed using an independent *t*-test. For concurrent validity, the correlation between the K-PPAQ and K-GPAQ were analyzed using Pearson’s correlation coefficient. The reliability of the K-PPAQ was assessed using the Guttman split-half coefficient to examine the homogeneity of the items. The Guttman Split Half Reliability coefficient is an adaptation of the Spearman–Brown coefficient, but one which does not require equal variances between the two split forms. The best case is both halves containing highly inter-correlated items. During the statistical analysis for the split half reliability (Spearman and Brown formula and Guttmann’s formula) and Cronbach’s Alpha reliability, the SPSS Statistics 20 software (IBM, Armonk, NY, USA) was used for calculations.

## 3. Results

### 3.1. Participants’ Demographics

The mean participant age was 32.87 years old, with 59 participants (16.3%) below the age of 30, 287 (79.1%) in their 30s, and 17 (4.6%) in their 40s or older. While 178 (49.0%) had no children at the time of the study (excluding fetus), 185 (51.0%) had one or more. There were 39 (10.8%) who were high school graduates or lower, 84 (23.1%) were community college graduates, and 240 (66.1%) were four-year college graduates or higher. A total of 354 (97.5%) were married, while 9 (2.5%) were single. While 178 (49.0%) were housewives or students, 185 (51.0%) were employed. The mean gestation age was 28.61 weeks.

### 3.2. Content Validity of the PPAQ

Of the 36 items on the PPAQ, the date of survey, the first day of the last period, the estimated due date, and the two open-ended questions were excluded from the content validity testing; only the remaining 31 items were tested by a panel of experts. The content validity of the K-PPAQ was analyzed using the CVR suggested by Lawshe [22], where the cutoff of the Content Validity Ratio (CVR) is set to 1.0 for a panel of five experts. With reference to this, we set the cutoff of content validity to a CVR of 1.0, and 26 out of 31 items met the cutoff, based on which content validity was established. Further, based on a cutoff of below 0.50 for convergence and 0.75 or higher for consensus, six out of 31 items had a convergence of below 0.5. The coefficient of variation was calculated to determine whether the six items did not meet the cutoff for CVR and the degree of convergence, and none of them had a value of above the cutoff of 0.8, based on which all of the items were included in the K-PPAQ (Table 1). However, in the pilot test on pregnant women, there were opinions that the items “slowly walk or remain standing while holding an object (a weight of about 4 L or greater)” and “quickly walk or remain standing while holding an object (a weight of about 4 L or greater)” should present specific examples of objects that weigh 4 L for better understanding; thus, we revised the phrasing to “slowly walk or remain standing while holding an object (a weight of about 4 L or greater; for example: about 2.6 water bottles of 1.5 L)” and “quickly walk or remain standing while holding an object (a weight of about 4 L or greater; for example: about 2.6 water bottles of 1.5 L).”

### 3.3. Amount of Physical Activities in the PPAQ 

The median and mean PPAQ scores for the total amount of physical activity were 20.32 MET-h × d^−1^ and 22.84 MET-h × d^−1^, respectively. The median and mean scores for total activity (light-intensity activity or greater) were 17.20 MET-h × d^−1^ and 18.99 MET-h × d^−1^, respectively. By the intensity of physical activity, the median and mean scores for sedentary activity were 2.70 MET-h × wkd^−1^ and 3.86 MET-h × d^−1^, respectively. The median and mean scores for light-intensity activity were 13.15 MET-h × d^−1^ and 13.67 MET-h × d^−1^, respectively. The median and mean scores for moderate-intensity activity were 3.16 MET-h × d^−1^ and 4.97 MET-h × d^−1^, respectively. The median and mean scores for vigorous-intensity activity were 0.00 MET-h × d^−1^ and 0.36 MET-h × d^−1^, respectively. By type of activity, the median scores were 6.90 MET-h × d^−1^ for household tasks/caregiving, 0.19 MET-h × d^−1^ for occupational, 0.52 MET-h × d^−1^ for sports/exercise, and 1.73 MET-h × d^−1^ for transport, and the mean scores were 18.12 MET-h × d^−1^ for household tasks/caregiving, 4.85 MET-h × d^−1^ for occupational, 1.14 MET-h × d^−1^ for sports/exercise, and 2.34 MET-h × d^−1^ for transport. The median and mean scores for inactivity were 4.12 MET-h × d^−1^ and 4.58 MET-h × d^−1^ (Table 2).

### 3.4. Comparison of Amount of Physical Activities between the Primipara and Multipara Women in the PPAQ

In the construct validity testing, the physical activities on the PPAQ were compared based on the presence of other children, and there were statistically significant differences in the total activity between the primipara and multipara women. There were significant differences in the total activity (t = −4.56, *p* < 0.001), total activity (≥light-intensity activity) (t = −5.80, *p* < 0.001), sedentary (t = 4.08, *p* < 0.001), light activity (t = −4.21, *p* <0 0.001), moderate activity (t = −5.75, *p* < 0.001), household tasks/caregiving (t = −6.13, *p* < 0.001), and inactivity (t = 4.55, *p* < 0.001). However, there were no significant differences in vigorous activity (t = 0.56, *p* = 0.572), occupational (t = −0.11, *p* = 0.887), sports/exercise (t = 1.65, *p* < 0.098), and transportation (t = −1.14, *p* = 0.242) between the primipara and multipara women (Table 3).

### 3.5. Concurrent Validity Testing

Concurrent validity was tested by examining the correlation between the PPAQ and GPAQ, and there were statistically significant correlations in all the domains, with the exception of the vigorous activity domain in the PPAQ and the high-intensity activity domain in the GPAQ (Table 4).

### 3.6. Reliability Testing

In this study, we computed the Guttman half-split reliability coefficient to examine the homogeneity of the items, and the overall Guttman lambda was 0.84.

## 4. Discussion

This study adapted and validated the PPAQ into Korean, and the results of the study are discussed in the following paragraphs.

First, the content validity of the K-PPAQ was tested by relevant experts, and the panel found that 26 out of the 31 items on the K-PPAQ meet the CVR cutoff of 1.0 suggested by Lawshe [22] and the cutoff of 0.60–1.00 suggested by Polit, Beck, and Owen [23]. The coefficient of variation was computed for the remaining six items that did not meet the cutoff for CVR and convergence, and the coefficient of variation exceeded the cutoff of 0.8, confirming that the items on the K-PPAQ are suitable to measure physical activity in pregnant women. These results re-confirm that various physical activities, including household tasks/caregiving, occupation, sports/exercise, transport, and rest are significantly relevant to measuring the physical activity level and lifestyle in pregnant women in Korea. Particularly, as existing physical activity questionnaires are mostly centered on high-intensity sports and fail to reflect the differences in the level of physical activity among pregnant women, this questionnaire is significant in that it can examine physical activities among pregnant women in detail. Therefore, subsequent studies should accurately understand physical activities in pregnant women using the K-PPAQ validated in this study and implement interventions accordingly.

Second, this study compared physical activities measured with PPAQ between pregnant women with or without children to test the construct validity of the questionnaire. The results showed that the two groups statistically significantly differed in the total activity and total activity (≥light-intensity activity), based on which the construct validity of the K-PPAQ was established. Whereas Chasan-Taber et al. [16] established construct validity on the same group of participants at different points in time, we utilized known-group comparison since pregnant women are at-risk study participants who may, along with their families, consider taking a survey during pregnancy stressful and therefore may be reluctant to participate. The fact that we were still able to establish the construct validity of the K-PPAQ suggests that the K-PPAQ is a valid instrument for measuring physical activities in pregnant women in Korea.

Moreover, the median value of total activity (≥light-intensity activity) in this study was 13.15 MET-h × d^−1^. This is a lower value than the first PPAQ (25.2 MET-h × d^−1^) and the second PPAQ (22.2 MET-h × d^−1^) in the study by Chasan-Taber et al. [16]. In our study, the median values for sedentary activity, light activity, moderate activity, and vigorous activity were 2.70, 13.15, 3.16, and 0.00 MET-h × d^−1^, respectively, while the median values were 9.3, 14.6, 10.4, and 0.0 MET-h × d^−1^, respectively, in the study by Chasan-Taber et al. [16]. The amount of physical activity during pregnancy is estimated to vary by culture, just like the previous study [8,24,25] that stated that there was a difference depending on not only personal aspects such as age and level of education, but also cultural, ethical, and socioeconomic levels as well. In South Korea, there are seven ancient traditional virtues that a pregnant woman is expected to keep (the Chil Tae-do) [26], even to this day. These virtues include avoiding carrying heavy loads, refraining from talking too much or crying, avoiding dangerous places, reading books quietly, writing poetry and listening to music, avoiding leaning or standing on one foot, keeping close to and appreciating valuable objects, and practicing abstinence during pregnancy. South Korea has traditionally emphasized being careful of physical exercise and movement during pregnancy. Therefore, Korean pregnant women rarely engaged in vigorous-intensity activity, which supports the literature findings that Korean pregnant women are less likely to participate in intense activities [9,27].

This is also attributable to the fact that there is a strong cultural belief in Korea that vigorous exercise during pregnancy can strain the mother’s body; thus, healthcare providers recommend light, as opposed to vigorous, exercise. In particular, we found that Korean pregnant women engage in less physical activity than their American counterparts, as pregnant women in the US do about two times more moderate-intensity activity. The amount of physical activity of pregnant women in Korea tends to be lower than the amount of physical activity shown in studies regarding pregnant women in China and Taiwan, countries of the same Asian region [28]. These results suggest that applying diverse pregnancy physical activities based on the K-PPAQ are needed. Although it varies depending on national beliefs and characteristics pertinent to pregnancy, this may be due to the fact that the Korean society prioritizes adequate nutritional intake and rest and believes that physical activity has an adverse impact on the fetus [9,27]. However, exercising during pregnancy helps maintain a stable psychological state. Exercise not only alleviates the stress, anxiety, and depression symptoms that occur during pregnancy [2], but also reduces the duration and pain of labor and facilitates postpartum recovery [7]. A meta-analysis of the impact of physical activity on pregnant women reported that moderate-intensity activities lower excessive pregnancy weight gain, pregnancy diabetes, and postpartum depression [11]. Therefore, it is necessary to provide suitable exercise programs for pregnant women in South Korea.

By type of physical activity, the median values for household tasks/caregiving, occupational, and exercise were 6.90, 0.19, and 0.52 MET-h × d^−1^, respectively, and those in the study by Chasan-Taber et al. [16] were 11.3, 10.6, and 1.6 MET-h × d^−1^, respectively. There was a marked difference in the level of occupational activity, and this is speculated to be due to the fact that 49% of our participants were housewives or students. Particularly, pregnant women in Korea spent the greatest amount of time on household tasks/caregiving, followed by inactivity, indicating that pregnant women spend less time on activities other than household tasks/caregiving.

Third, we tested the concurrent validity of the K-PPAQ by examining its correlation with the K-GPAQ, and all domains of the K-PPAQ were significantly correlated with the high-intensity and moderate-intensity activities of the GPAQ. The K-PPAQ and K-GPAQ seem to be similar, as they are strongly correlated. Unlike the GPAQ, however, the PPAQ classifies the physical activities of pregnant women based on intensity and type; thus, it is a more valid instrument for measuring physical activities among pregnant women. However, similar to the findings of the Chasan-Taber et al. [16], pregnant women in our study rarely engaged in vigorous exercise, and there was no correlation between the two questionnaires in vigorous exercise. Findings indicating that vigorous exercise in early pregnancy can cause early miscarriage, prolonged high-intensity exercise can increase the risk of acute fetal hypoxia, and exercise that excessively strains the abdomen can cause increased intraabdominal pressure and thus affect the fetus [1] suggest that it can be difficult for pregnant women to consider vigorous exercise. Nevertheless, we did not remove the items for vigorous exercise, because the PPAQ was originally developed to detect sedentary, light, moderate, and vigorous activities in wide-ranging areas (household tasks/caregiving, occupational, sports/exercise) in ethnically diverse pregnant women [16]. In addition, because the PPAQ calculates the total physical activity, which encompasses a variety of types of physical activities, there is a limitation in excluding some of the activities. Further, we used the Guttman’s lambda through the split-half method to examine the reliability of the K-PPAQ; the Guttman’s lambda for the K-PPAQ was calculated to be 0.84, indicating a high reliability.

This study established the reliability and validity of the K-PPAQ for examining the types and intensities of physical activities among pregnant women. With this questionnaire, pregnant women in Korea should also be given individualized counseling about physical activities during pregnancy so as to promote their participation in various customized education and programs that would facilitate physical activity. Subsequent studies should analyze the level of physical activities among pregnant women using the PPAQ and develop relevant education, physical activity plans, and programs tailored to the gestational age of pregnant women.

## 5. Conclusions

This study aimed to adapt and validate the PPAQ into Korean so as to provide foundational data for developing physical activity interventions for pregnant women. After testing the content validity of the PPAQ, all the items were included in the Korean version. In this study, the Korean version of the PPAQ is a valid and reliable instrument for measuring the physical activity level in Korean pregnant women. The study included women of all gestational age. However, in Korea, it was difficult to obtain even data samples of pregnant women for each gestational age. This is mentioned as one of the limitations of the study.

## Figures and Tables

**Table 1:** Content validity verification.

Item	CVR ^1^	Convergence	Consensus	Coefficient of Variation
Preparing meals (cook, set table, wash dishes)	1.0	0.0	1.0	0.0
Dressing, bathing, feeding children while you are sitting	1.0	0.0	1.0	0.0
Dressing, bathing, feeding children while you are standing	1.0	0.0	1.0	0.0
Playing with children while you are sitting or standing	1.0	0.0	1.0	0.0
Playing with children while you are walking or running	1.0	0.0	1.0	0.0
Carrying children	1.0	0.0	1.0	0.0
Taking care of an older adult	0.6	0.5	0.8	0.1
Sitting and using a computer or writing, while not at work	1.0	0.0	1.0	0.0
Watching TV or a video	1.0	0.0	1.0	0.0
Sitting and reading, talking, or on the phone, while not at work	1.0	0.0	1.0	0.0
Playing with pets	1.0	0.0	1.0	0.0
Light cleaning (make beds, laundry, iron, put things away)	1.0	0.0	1.0	0.0
Shopping (for food, clothes, or other items)	1.0	0.0	1.0	0.0
Heavier cleaning (vacuum, mop, sweep, wash windows)	1.0	0.0	1.0	0.1
Mowing lawn while on a riding mower	0.6	0.5	0.8	0.2
Mowing lawn using a walking mower, raking, gardening	0.6	0.5	0.8	0.2
Walking slowly to go places (such as to the bus, work, visiting) (not for fun or exercise)	1.0	0.0	1.0	0.0
Walking quickly to go places (such as to the bus, work, or school) (not for fun or exercise)	1.0	0.0	1.0	0.0
Driving or riding in a car or bus	1.0	0.0	1.0	0.0
Walking slowly for fun or exercise	1.0	0.0	1.0	0.0
Walking more quickly for fun or exercise	1.0	0.0	1.0	0.0
Walking quickly up hills for fun or exercise	1.0	0.0	1.0	0.1
Jogging	1.0	0.0	1.0	0.0
Prenatal exercise class	1.0	0.0	1.0	0.0
Swimming	1.0	0.0	1.0	0.0
Dancing	1.0	0.0	1.0	0.0
Sitting at working or in class	1.0	0.0	1.0	0.0
Standing or slowly walking at work while carrying things (heavier than a 1-gallon milk jug)	0.6	0.5	0.8	0.2
Standing or slowly walking at work not carrying anything	0.6	0.5	0.8	0.2
Walking quickly at work while carrying things (heavier than a 1-gallon milk jug)	1.0	0.0	1.0	0.2
Walking quickly at work not carrying anything	1.0	0.0	1.0	0.0

^1^ CVR = Content Validity Ratio.

**Table 2:** Descriptive analysis of Pregnancy Physical Activity Questionnaire (PPAQ) on participants.

(Unit: MET-h × MET^−1^)									
Variables	25th	Median	75th	Mean	Std. Deviation	Skewness	Std. Error	Kurtosis	Std. Error
Summary activity scores									
Total activity	15.53	20.32	27.60	22.84	11.39	1.84	0.12	5.58	0.25
Total activity (light and above)	11.92	17.20	23.59	18.99	11.55	1.78	0.12	5.36	0.25
By intensity									
Sedentary activity	1.05	2.70	4.90	3.86	3.23	1.13	0.12	0.58	0.25
Light intensity	8.79	13.15	17.75	13.67	7.26	0.93	0.12	2.47	0.25
Moderate intensity	1.19	3.16	5.86	4.97	4.01	2.69	0.12	9.11	0.25
Vigorous intensity	0.00	0.00	0.23	0.36	0.83	3.28	0.12	1.53	0.25
By type									
Household/caregiving	3.59	6.90	17.53	18.12	11.77	3.69	0.12	2.87	0.25
Occupation	0.00	0.19	9.60	4.85	4.09	2.62	0.12	8.52	0.25
Sports/exercise	0.06	0.52	1.45	1.14	1.59	2.46	0.12	7.80	0.25
Transportation	0.96	1.73	3.16	2.34	2.35	2.54	0.12	8.77	0.25
Inactivity	1.26	4.12	6.40	4.58	3.55	1.05	0.12	0.57	0.25

PPAQ = Pregnancy Physical Activity Questionnaire.

**Table 3:** Comparison of PPAQ according to group.

Variables	Primipara M ± SD	Multipara M ± SD	t	df	*p*	Mean Difference	Std. Error Difference	95% Confidence Interval of the Difference	
								Lower	Upper
Total activity	19.09 ± 7.49	24.74 ± 12.53	−4.56	361	<0.001	−5.64	1.23	−8.08	−3.21
Total activity (light and above)	14.23 ± 7.51	21.40 ± 12.47	−5.80	361	<0.001	−7.16	1.23	−9.59	−4.73
Sedentary activity	4.81 ± 3.48	3.38 ± 2.99	4.08	361	<0.001	1.43	0.35	0.74	2.12
Light Intensity	11.43 ± 6.53	14.81 ± 7.36	−4.21	361	<0.001	−3.37	0.78	−4.92	−1.86
Moderate Intensity	2.52 ± 2.89	6.22 ± 6.76	−5.75	361	<0.001	−3.37	0.78	−4.96	−2.43
Vigorous Intensity	0.39 ± 0.87	0.34 ± 0.82	0.56	361	0.572	0.05	0.09	−0.13	0.54
Household/caregiving	4.50 ± 3.73	25.10 ± 37.11	−6.13	361	<0.001	−20.59	3.35	−27.19	−13.99
Occupation	4.73 ± 4.84	4.91 ± 18.25	−0.11	361	0.887	−0.17	1.67	−3.34	3.11
Sports/exercise	1.34 ± 1.65	1.04 ± 1.56	1.65	361	0.098	0.02	0.17	−0.05	0.64
Transportation	2.14 ± 2.23	2.44 ± 2.41	−1.14	361	0.242	−0.29	0.25	−0.81	0.22
Inactivity	5.74 ± 3.64	3.99 ± 3.35	4.55	361	<0.001	1.75	0.38	0.99	2.50

Df = Degree of freedom; PPAQ = Pregnancy Physical Activity Questionnaire.

**Table 4:** Correlation between PPAQ and GPAQ.

Variables	X1	X2	X3	X4	X5	X6	X7	X8	X9	X10
X1: PPAQ-Total activity	1									
X2: PPAQ-Total activity light intensity and above	0.96 *	1								
X3: PPAQ-Sedentary activity	−0.92 *	−0.89 *	1							
X4: PPAQ-Light intensity	0.84 *	0.88 *	−0.60 *	1						
X5: PPAQ-Moderate intensity	0.82 *	0.85 *	−0.65 *	0.61 *	1					
X6: PPAQ-Vigorous intensity	0.06	0.03	0.09 *	0.11 *	0.07	1				
X7: GPAQ-Walking time	0.66 *	0.60 *	−0.49 *	0.54 *	0.85 *	0.02	1			
X8: GPAQ-Sedentary time	−0.63 *	−0.49 *	0.65 *	−0.69 *	−0.69 *	−0.06	−0.62 *	1		
X9: GPAQ-Vigorous intensity activity	0.02	0.06	0.06	0.05	0.01	0.01	0.02	−0.08	1	
X10: GPAQ-Moderate intensity activity	0.63 *	0.78 *	−0.83 *	0.67 *	0.81 *	0.08 *	0.81 *	0.48 *	0.40 *	1

GPAQ = Global Physical Activity Questionnaire; PPAQ = Pregnancy Physical Activity Questionnaire; * = *p* < 0.05.
